# The Influence of Socioeconomic and Environmental Determinants on Health and Obesity: A West Virginia Case Study

**DOI:** 10.3390/ijerph6082271

**Published:** 2009-08-19

**Authors:** Anura Amarasinghe, Gerard D’Souza, Cheryl Brown, Hyungna Oh, Tatiana Borisova

**Affiliations:** 1 Centre for the Built Environment and Health, School of Population Health, The University of Western Australia, 35 Stirling Highway, Crawley, Western Australia 6009, Australia; E-Mail: AAmarasinghe@meddent.uwa.edu.au; 2 Division of Resource Management, West Virginia University, P.O. Box 6108, Morgantown, WV 26505-6108, USA; E-Mail: Cheryl.Brown@mail.wvu.edu; 3 College of Business and Economics, West Virginia University, P.O. Box 6025, Morgantown, WV 26505-6108, USA; E-Mail: Hyungna.Oh@mail.wvu.edu; 4 Food and Resource Economics Department, University of Florida, McCarty Hall A, P.O. Box 110240, Gainesville, FL 32611-0240, USA; E-Mail: borisova.tancha@gmail.com

**Keywords:** health, obesity, endogeneity, human capital, land use, Appalachia

## Abstract

A recursive system of ordered self assessed health together with BRFSS data were used to investigate health and obesity in the Appalachian state of West Virginia. Implications of unobserved heterogeneity and endogeneity of lifestyle outcomes on health were investigated. Obesity was found to be an endogenous lifestyle outcome associated with impaired health status. Risk of obesity is found to increase at a decreasing rate with per capita income and age. Intervention measures which stimulate human capital development, diet-disease knowledge and careful land use planning may improve health and obesity outcomes in Appalachia in particular and rural America in general.

## 1. Introduction

Over the past few decades, a combination of economic, structural, and behavioral changes have had profound impacts on lifestyle behaviors, with often adverse impacts on health. Overweight and obesity are some non-contagious health outcomes that have escalated, mostly due to lifestyle behaviors. Obesity is defined in terms of Body Mass Index (BMI), which is a measure of body fat content, and also a function of both height and weight. According to National Institute of Health (NIH) guidelines, individuals whose body mass index (BMI) is greater than or equal to 30 kg/m^2^ are considered to be obese, and those with a BMI between 25–29.9 kg/m^2^ are considered overweight. Overweight and obesity increase the risk of having most prevailing diseases, including diabetes, cardiovascular diseases and cancer [1–3]. The consequences of obesity are manifested in soaring health care costs, which, in the U.S. are estimated to be $117 billion/year, with approximately 300,000 direct and indirect deaths per year attributable to the problem [4].

West Virginia (WV) is a U.S. state—the only one that lies wholly within Appalachia—that is both rural and economically lagging, with one of the highest obesity rates in the country. WV is ranked as the third highest obesity prevalence state, next to Mississippi and Alabama; in addition, WV has the third lowest income per-capita among U.S. states [5]. The obesity rate has increased in virtually all WV counties over the past decade, with the highest prevalence found in the southern and western portions of the state [6]. The objective of this study is to examine the causes and consequences of obesity on health in West Virginians, with implications for other predominantly rural areas of the U.S. This study also investigated the empirical implications of unobserved heterogeneity and endogeneity of the health-lifestyle interrelationship.

## 2. Background

The seminal contributions of the household production framework and theory of the allocation of time [7,8] showed that households can combine time and resources to produce a commodity of good health that yields utility. Utility is a measure of the relative satisfaction derived from the consumption of various goods and services. The underlying theory of the household production framework assumes that individuals do not receive utility or satisfaction directly from the goods that they purchase from the market. Instead, it is only when market goods are combined with time inputs that utility-providing outputs are produced. This framework has been extended to investigate investments in health capital influenced by the consumer’s time and market goods such as medical care, diet, exercise, recreation and housing, as well as exogenous socioeconomic and demographic characteristics [9,10]. An individual’s health can be influenced by both observed (e.g., lifestyle behaviors such as smoking, eating and drinking) and unobserved factors (e.g., unobserved genetic, hormonal and biochemical factors) [10–13].

Even though the measures of health are multifaceted, self-assessed health (SAH) has been extensively used as an indicator of individual health [12,14–16]. A multivariate analysis of the British Health and Lifestyle Survey (HALS) showed that discrete indicators of lifestyle behaviors such as sleeping well, exercising, and not smoking may have a positive effect on the probability of reporting excellent or good SAH [16]. A cardinal measure of health status, self reported number of physically healthy days [17] indicated that health inequalities in the U.S. population are prominent in elderly, less educated, female and low-income groups. It has been shown that improved diet-disease knowledge can promote health by choosing better lifestyle behaviors related to diet, smoking, exercise, alcohol consumption, sleep, weight (relative to height) and stress [12].

Obesity, a result of individuals’ lifestyle behaviors, has been found to have significant effects on an individual’s health and longevity, as well as on the economy as a whole [18]. Several studies contend that technological change associated with relatively cheap food prices and sedentary behaviors at work and in daily life contributes to obesity [18,19]. In contrast to the developing world, most postindustrial and redistributive societies, such as the U.S., entail work with little exercise. As a result, people must pay for undertaking, rather than being paid to undertake, physical activity. Payment is mostly in terms of forgone leisure, because leisure activity for weight control must be substituted for weight control by physical exertion at work [18]. However, the wage penalty or the opportunity cost of time, time-use decisions, and health of a family have become even important issues in postindustrialized societies as more and more women participate in the labor force. In the U.S., the labor force participation rate of women with young children (under 6 years of age) increased from 39% in 1975 to 62% in 1996 [20]. Increased participation of women in the labor force has reduced time available for non-market household activities and motivated people to consume relatively cheap highcaloric foods leading to overweight and obesity [21]. Studies have also suggested that increased consumption high-caloric foods is further triggered by the abundant availability of fast food outlets which offer relatively inexpensive food menus [19,20]. In line with economic factors, others argued that smoking, unemployment, and job strenuousness were other factors that could lead to obesity [21,22].

Wealth and poverty have profound effects on diet structure, nutrition and health. Investigation of health response to changes in the economic environment indicate that smoking and obesity increases when the economy strengthens, while, simultaneously, physical activity is reduced and diet becomes unhealthy [22]. In higher income nations, cost per unit of food energy is low, such that those nations are associated with high-energy intakes. Accordingly, people in higher income nations consume more added sugars and fats than those in low-income nations. In addition, low-income consumers within rich nations consume lower quality diets than do higher income consumers [23]. Indeed, poverty and food insecurity are associated with lower food expenditures, low fruit and vegetable consumption, and lower-quality diets [24]. Thus overweight and obesity is considerably higher among racial/ethnic minorities and among lower income groups with the least amount of education [25–27].

In addition to economic growth factors, social capital contributes to better health through the diffusion of knowledge about health promotion, maintenance of healthy behavioral norms through informal social control and access to local services and amenities [28–31]. Findings also suggest that lifestyle behaviors are culturally driven, so that individual self image and social interactions could play a role in determining one’s body weight [32,33]. Being overweight can also have a negative impact on one’s self image and may contribute to the rising social phenomenon of divorces [18]. Poor body image has the potential to affect physical and mental health creating psychosocial distress and thus lower academic performance [34,35]. In more recent times, urban sprawl, characterized by low density residential development, low employment density, and poor street connectivity are associated with less walking and bicycling and with more automobile travel than denser communities, thereby promoting increased isolation and/or decreased social cohesion. This has profound effects on low levels of physical activity, obesity and public health directly [36–39]. One study suggested that each additional hour spent in a car per day was associated with a 6% increase in the likelihood of obesity [38]. With this as a backdrop, we set out to investigate how socioeconomic and physical environments impact obesity, health, and quality of life in rural settings.

## 3. Methodology

An individual’s health can be affected by a complex set of observed and unobserved heterogeneous or variable factors. Thus, lifestyle behaviors, which enter into the health production function are arguably endogenous in nature, as they can be highly correlated with the error term or unobserved factors related to health [13,16]. In this study we hypothesized that the lifestyle outcome of obesity, which is related to weight and health status, may be correlated to an unobservable variable relegated to the error term. The failure of epidemiological analyses to account for unobserved variation can give biased estimates in the socioeconomic-health relationship [16]. In order to address such endogeneity bias, a two stage recursive approach was used for this study. In this two stage estimation process, an ordered latent-class variable of self-assessed health is considered to be explained by the individual’s socioeconomic, demographic and environmental covariates. Denoting individual *i*’s unobserved latent health status as $H_{i}^{*}$, individual self assessed health can be written as: $H_{i}^{*}=\phi^{\prime }\;L_{i}^{*}+\omega^{\prime }\;X+u_{i}$, where *u_i_* ∼ (0,1). The vectors L^*^ and *X* represent lifestyle behaviors and other socioeconomic, demographic and environmental characteristics, respectively. The individual’s health status, *H_i_*, is equal to *k*, if *μ_ik_* < $H_{i}^{*}$ ≤ *μ_ik_*_+1_ where the parameter *k* ∈ [1,2,3] represents three self-assessed health categories: “poor,” “fair,” and “good”. The parameter *μ_ik_*, which varies from −∞ to +∞, denotes the unknown threshold levels of health categories that are to be estimated together with parameters ϕ and ω. Thus, the probability, *P*, of having a certain health status can be defined as:

(1) $$P\left[H_{i}=1|X,L\right]=\Phi (\mu_{k}-\phi^{\prime }\;L_{i}^{*}-\omega^{\prime }\;X)$$

(2) $$P\left[H_{i}=2|X,L\right]=\Phi (\mu_{k+1}-\phi^{\prime }\;L_{i}^{*}-\omega^{\prime }\;X)-\Phi (\mu_{k}-\phi^{\prime }\;L_{i}^{*}-\omega^{\prime }\;X)$$

(3) $$P\left[H_{i}-3|X,L\right]=1-\Phi (\mu_{k+1}-\phi^{\prime }\;L_{i}^{*}-\omega^{\prime }\;X)$$

where Φdenotes the cumulative distribution function (CDF) of the standard normal distribution. Since the vector of lifestyle behaviors, $L_{i}^{*}$, is assumed to be endogenous to the system, it could be correlated with unobserved factors affecting one’s self-assessed health (SAH). Endogeneity bias was overcome by a recursive estimation process in which the first stage predictions of lifestyle behavior were incorporated into the self-assessed health variable. The fully recursive system can then be specified as:

(4) $$H_{1i}^{*}=\phi^{\prime }\;L_{i}^{*}+\omega^{\prime }\;X_{1}+u_{1i},\;\;u_{1i}∼\left(0,\;\sigma_{u_{1i}}^{2}\right)$$

(5) $$L_{2i}^{*}=\omega^{\prime }\;X_{2}+u_{2i},\;u_{2i}∼\left(0,\;\sigma_{u_{2i}}^{2}\right)$$

where E(*u*_1_*_i_u*_2_*_i_*) = σ_12_, *i* = 1,2,...,*I;* E(*u_ji_u_j′__i′_*) = 0 for *j* = 1,2 *j′* = 1,2, *i* ≠ *i′* ; and $L_{2i}^{*}$ is another latent-class variable of lifestyle behaviors. For example, obesity, which represents an individual’s weight status, was considered a latent-class dependent variable in equation (5) above. As stated previously, obesity was an endogenous factor in the individual self assessed health equation (4) above. Therefore, it is assumed that correlation of the error terms between equations is σ_12_ and is zero for the different observations of two equations. Equations are identified through exclusion parameter restrictions between them. Unobserved variation of lifestyle choices is tested through the procedures outlined by Smith 1987 [40]. Accordingly we also estimated an original self assessed health equation, while incorporating the residuals obtained from the first stage estimation of obesity:

(6) $$H_{1i}^{*}=\phi^{\prime }\;L_{i}^{*}+\omega^{\prime }\;X_{1}+\alpha u_{2i}+{ɛ}_{1i}\;\;\text{where}\;u_{1i}=u_{2i}+{ɛ}_{1i}$$

The hypothesis *H*_0_: α = 0 implies that obesity is weakly exogenous to the health equation (6) above. Endogenity of obesity is tested by the simplified likelihood ratio test (SLR) [41] and the Hausman test [42]. This two stage estimation process allows correcting unobserved variations which may affect both health and lifestyle behaviors and is also expected to improve the efficiency of estimates.

## 4. Data and Estimation Procedure

Individual data for the state of WV are compiled from the Behavioral Risk Factor Surveillance System (BRFSS), year 2003 micro data files (the most recently available when this analysis was conducted) that investigated adult health behavior across the state [43]. BRFSS is a monthly telephone survey conducted by the Centers for Disease Control and Prevention (CDC) that allows states to monitor health behaviors among their adult population (18 years of age or older). The BRFSS was begun in 1984 with 15 participating states and has monitored obesity since that time, expanding to 52 states and territories in 1997 [43]. Data for the BRFSS survey were collected from a random sample of one adult per household through a computer-assisted telephone interviewing method with an overall response rate of 42%. County specific land use and other socioeconomic variables were obtained from the U.S census bureau and the Appalachian regional commission [44,45].

The variable definitions and summary statistics are presented in Tables 1 and 2. OBESE and OGENHLTH are categorical dependent variables in the recursive system represented by equations (4) and (5). OBESE is a binary dependent variable which indicates whether a person is obese (equal to 1) or not (equal to 0). Individuals whose body mass index (BMI) is greater than or equal to 30 kg/m^2^, are considered to be obese. OGENHLTH is an ordered latent-class dependent variable which indicates the individual’s ordered self-assessed health (SAH) responses of “good”, “fair” or “poor”.

Level of education (LEDUCA) is an ordered categorical explanatory variable which varies from 0 to 5. The resulting six educational categories are: (0) never attended school or kindergarten, (1) attended elementary school, (2) attended some high school, (3) high school graduate, (4) attended college, and (5) college graduate. DSEX is a gender dummy for which female is the base category (=0). Hispanics (HISP), white non-Hispanics (WNONH), black non-Hispanics (BNONH) and multicultural non-Hispanics (MNONH) are dummy variables representing the ethnic composition of the sample. Per capita income (PINC) is created by considering the mid-points of the income categories to which an individual belongs in the sample. Individuals are assumed to belong to four income categories ranging from less than $15000; $15,000 < $35,000; $35,000 < $ 50,000; and over $50,000. Individuals who have incomes equal to or greater than $50,000 are assumed to have a per capita income of $50,000. In order to investigate a nonlinear impact of per capita income (PINC), income squared (INCSQ) is also added as an explanatory variable to the model. Employed (EMPLOYD), student (STUDENT), retired (RETD) and other (OTHERE) are dummy explanatory variables which represent the employment status of individuals. Other employment, which served as the reference category, includes individuals who are unable to work or were out of work for about one year.

Widowed (WIDOW), married or cohabiting (MALT), divorced or separated (DIVSEP) and never married (NMARRI) represent the marital status of individuals. Sedentary (SEDENT) is a dummy variable which captures physical activity, with respondents who report no moderate or vigorous physical activity or exercise considered to be sedentary or physically inactive. SMOKING is a dummy variable which takes the value 1 if an individual ever smoked 100 cigarettes in his/her lifetime and now smokes every day or some days. SMOKING takes the value 0 if an individual does not smoke now. HCARE, RHEART, RASTHMA, RFDRHV are dummy variables which indicate whether an individual possesses a health care plan, is at risk of having heart ailments, is at risk of having asthma problems and is at risk of being a heavy consumer of alcohol, respectively. Risk of heavy alcohol consumption is determined by whether a male respondent has more than 2 drinks per day, or a female respondent has more than 1 drink per day. FRTINDX is an ordered categorical variable which describes fruit and vegetable consumption of respondents. The fruit and vegetable consumption frequencies, ordered from 1 to 4, represent whether a respondent’s consumption is less than 1 serving per day, 1 to less than 3 servings per day, 3 to less than 5 servings per day, or 5 or more servings per day.

Average travel time to work in a county (TRVT) is an explanatory variable that is included to capture the potential influence of the built environment on obesity. TRVT was computed by using information from the 2000 U.S. Census [44]. A county specific dummy variable which indicates the economic status of a respondent’s county, i.e., whether the county is economically depressed or not is included (DDISTD) [45]. Using the Appalachian Regional Commission classification scheme, county economic status is depressed if the county’s three-year average unemployment rate is at least 1.5 times the national average, per capita market income is no greater than two-thirds of the national average, and the poverty rate is at least 1.5 times the national average; or the county has at least twice the national poverty rate and meets the criteria for either the unemployment or the income indicator. We believe that to a certain degree, this variable represents the individual or neighborhood socioeconomic status. Even though we investigated the impact of county specific natural and built environment with a set of principal components, we excluded those from the regressions since there was no significant impact on the results.

In carrying out the estimations, equation (5) which represents individual weight status was estimated in the first stage. In the second stage, equation (4), the ordered self-assessed health, which is incorporated with the predicted values of the first stage, was estimated. As previously stated, the equations of the system are identified through nonlinearity restrictions imposed on the squared terms of income and age parameters. These restrictions also help us to maintain the equal slope assumption of the ordered logit procedure.

## 5. Results

### 5.1. Preliminary Observations

Preliminary statistics indicated that the mean BMI of West Virginian is around 27.42 kg/m^2^, with a 25% prevalence of obesity. About 73% participants were reported to have a good health status, while 17% and 10% of them recorded fair and poor health, respectively. A significant proportion, more than 75%, had finished high school education. In terms of ethnicity, the highest number of participants were white non hispanic (91%) followed by multicultural non hispanics (4.5%). Even though above 50% of participants were either married or cohabited, a fairly high percentage of divorce (20%) can also be observed. Similar to education, above 75% reported having health care coverage. While 26% of the participants were smokers, 13% of them were sedentary. About 37% and 9% of participants, respectively, were reported to be at risk of having either heart or asthma problems. On average, about 23% were living in counties where distressed economic situations prevailed and commuted 25 minutes daily to work.

### 5.2. Empirical Estimation

The first stage binary logit estimations with the risk of being obese as the dependent variable (OBESE) are presented in Table 3. A binary probit estimation of obesity and ordered probit estimation of self assessed health were also conducted. However, these estimations showed similar directional impacts as the logit estimations and are not further discussed here. The logit estimations showed that the level of educational attainment (LEDUCA) has a significant negative impact on an individual being obese. A one unit increase in educational level would lower the log odds of being obese by 0.184, while other variables in the model are held constant. Out of the ethnic categories, Hispanics (HISP) are less likely to be obese in comparison to the base category of other multicultural non-Hispanics. For a Hispanic, the log odds of being obese is lower by 0.86 units. The directional impact of AGESQ and INCSQ indicates that the probability of being obese increases at a decreasing rate with both age (AGE) and per capita income (PINC).

Other results show that students are less likely to be obese than their base counterparts (i.e., those who are unable to work or are out of work for more than one year). The expected probability of a student being obese is reduced by 0.8 units in log odds scale. None of the variables that represent marital status indicate a significant impact on the probability of an individual being obese. Considering risk behaviors, as expected, smoking (SMOKE) and a sedentary lifestyle (SEDENT) show opposite impacts on an individual being obese. While smoking negatively and significantly contributes to obesity, sedentary behavior positively and significantly contributes to obesity. Respondents who smoke reduce the log odds of being obese by 0.8 units. In contrast, respondents with sedentary lifestyles are more likely to be obese with log odds of 0.5 units. The fruit and vegetable consumption index (FRTVINDX) is also negatively correlated with obesity. As fruit and vegetable consumption increases, the log odds of being obese decrease by 0.1 units. Although the county economic situation (DDISTD) does not seem to show any significant impact on obesity, the average travel time to work (TRVT) positively contributes to the log odds of being obese. As average travel time to work (in minutes) increases by one unit, the log odds of being obese increase by 0.02 units. In comparison to the binary logit specification, the binary probit estimation yields similar directional impacts on the odds of being obese with regard to the variables discussed above. In addition, the binary probit specification shows that males (DSEX) are more likely to be obese than females.

Table 3 also presents the marginal probabilities of an individual being obese. It indicates that as the level of education increases, the probability of being obese decreases by 3%. Hispanics are 16% less likely to be obese compared to non-Hispanic ethnic groups. Even though per capita income (PINC) has a significant positive effect on the probability of an individual being obese, its marginal impact is shown to be very small. If the respondent is a student, the probability of being obese is reduced by about 16%. As age increases, the marginal probability of being obese increases by 2%, at a decreasing rate. While the marginal impact of physical inactivity or a sedentary lifestyle (SEDENT) increases the risk of a person being obese by 9%, smoking reduces the risk of being obese by 15%. An increase in fruit and vegetable consumption significantly lowers the probability of a person being obese by 2%. A one minute increase in travel time increases the probability of being obese by 0.4%.

The simplified likelihood ratio (SLR) test statistic of the ordered logit models 2 and 3 in Table 4 equals 24.12. SLR equals −2(ln *L̄* – ln *L̂*) where *L̄* and *L̂* are log-likelihood values. This leads to the rejection of the exogenity of obesity when compared with the chi squared critical value, χ^2^ (0.05) = 3.84. This implies that obesity is an endogenous covariate that is correlated with unobserved factors affecting one’s self-assessed health. The Hausman test statistic, which examines the coefficient estimates associated with obesity, H = (2.8448–0.1798)^2^/[(0.1684)^2^–(0.0003)^2^] = 250.44, clearly rejects exogenity of obesity at 5% critical chi squared values.

Table 4 presents second stage ordered maximum likelihood logit estimates of self-assessed health (SAH) as explained by socioeconomic, demographic, risk behavior, and the respondent’s residential county-specific variables. The dependent variable (OGENHLTH) is an ordered latent-class variable which indicates the ordered self-assessed health (SAH) categories of “good,” “fair,” and “poor.” The variables, CONSTANT2 and CONSTANT1, are the estimated ordered logit for the adjacent level health category, “good” versus “fair” and “poor”, and “good” and “fair” versus “poor”, respectively, when the other covariates are evaluated at zero. For example, the log odds of “good” self-assessed health versus “fair” or “poor” for a female (i.e., DSEX evaluated at zero) is 2.04. The log odds of “good” and “fair” versus “poor” for a female is 3.62. The variable PREDOBE provides the predicted values of the first stage estimation for an individual being obese.

The socioeconomic variables educational attainment (LEDUCA) and income (PINC) significantly and positively raise the expected SAH. A unit increase in educational attainment would raise the expected SAH in ordered log odds scale by 0.2 units while the other variables in the model are held constant. Similarly, a $1,000 increase in income would raise the value of expected health by 0.03 units. Out of the covariates that describe employment status, those who are employed (EMPLOY) and retired (RETD) are the most likely to show good health. There is no significant contribution by gender to expected health. As age increases, expected SAH tends to decrease. The behavioral risk factors obesity, sedentary lifestyle and smoking negatively and significantly affect expected health. The expected SAH when one is obese (PREDOBE) decreases by 2.82 units in a log ordered scale. Similarly, having a sedentary lifestyle (SEDENT) would lower expected health by 0.60 units; and smoking (SMOKE) lowers expected health by 0.80 units. Obviously, respondents who are at risk of having heart ailments and asthma conditions are less likely to have good health. Risk of being a heart or asthma patient is found to lower the expected SAH in log ordered scale by about 0.80 units. Contrary to expectations, fruit and vegetable consumption does not show a significant impact on health. Lastly, respondents living in economically distressed counties are less likely to have good health. For a resident of an economically distressed county, the expected SAH in ordered log scale is lower by 0.48 units. None of the categories of marital status shows a significant difference for their expected SAH.

## 6. Discussion

In this analysis, a recursive system of multivariate ordered logit analysis of self assessed health (SAH) and a binary logit specification for risk of being obese were estimated in terms of socioeconomic, demographic and county specific socioeconomic indicators. Results showed that the level of education has a significant impact on the expected (SAH) health outcome and on the risk of being obese. Education positively and significantly contributes to better health, and significantly and negatively contributes to obesity. This reinforces results from previous studies [21,46,47] which also show that educational attainment has a negative impact on the probability of being obese. These findings seem quite relevant for US states like WV, where the educational differences across the area have been persistent over time [48]. Ordered logit estimations show that higher educational attainment significantly increases the probability of reporting better expected health outcomes. Previous findings also showed that individuals with lower educational levels have a significantly lower probability of reporting excellent or good health [16].

In terms of ethnicity, Hispanics are less likely to be obese than their non-Hispanic counterparts. Although this is contrary to previous findings, it could be quite possible in a WV setting. Over a 20 year period, the Hispanic share of the working class in the U.S. has increased three-fold, from 6% in 1980 to 20% in 2000, primarily due to immigration [44]. In WV, although the population with Hispanic origins has increased at a comparatively slower rate, from 0.5% in 1990 to 0.7% in 2000 [6] the sample considered for this study contained 2.1% Hispanics. A reasonable explanation for the results found in this study may be that the physical labor-intensive activities of this ethnic group, which constitutes a greater proportion of the “working class,” also contributes to their relative lack of obesity.

Previous research [21,46] also suggests that income negatively and significantly contributes to an individual being obese. In this study, we also looked at rates of change, and found that the risk of obesity increases at a decreasing rate with household income. The positive impact of income on health reinforces the fact that the “commodity” good health is a normal good.

Marital status does not significantly contribute either to obesity or to expected self-assessed health. This result is contrary to the previous finding [49] that married and widowed individuals have higher body mass index (BMI) and obesity odds, when compared to divorced and never-married individuals. Divorced individuals, in turn, have a lower weight outcome than those who have never married. The binary probit estimation shows that males are more likely to be obese than females. However, the impact of gender on obesity cannot be interpreted with great precision as its significance is not consistent across models. Nevertheless, one study [46] reveals that females tend to have more diet-disease knowledge than males and that such knowledge has a significant and negative effect on the probability of being obese.

The quadratic effect of age indicates that the probability of being obese increases with age but at a decreasing rate. Similar nonlinear age effects are also reported in previous research [21,47]. BMI and obesity appear to rise with age and then peak in the 50s, thereafter going down again for those in their 60s [49]. The negative coefficient of the AGE variable in the health equation suggests that as age increases, the probability of reporting good health decreases. Lee (1982) pointed out that health deteriorates with age, with the rate of health depreciation rising with age for middle-aged individuals.

Results from previous studies are equivocal in terms of risk behavior (i.e., smoking and sedentary lifestyles) impacts on obesity. For example, while some researchers [21] argue that smoking lowers the risk of being obese, others [49] claim that smoking increases the risk of obesity. Our results show that risky behaviors, including smoking and a sedentary lifestyle, and risk of having other health-impaired conditions such as heart disease and asthma are significantly and negatively correlated with an individual’s self-assessed health.

An interesting finding of this study is that commuting time to work is positively and significantly related to the risk of obesity. Similar to the urban sprawl hypothesis, residents of rural states like WV depend heavily on automobile travel when there are no economic development activities within their residential counties. Rural residents may travel to more distant areas not only for employment opportunities but also for their daily needs since supermarkets and grocery stores are sparsely distributed. Thus, increased reliance on automobile travel in association with less physical activity may lead to obesity and accompanying increased cardiovascular disease, diabetes and other health problems. In addition, respondents from economically distressed counties are more likely to have impaired health outcomes than respondents from economically advantaged counties.

Before discussing the policy implications, it is also necessary to note the limitations of this study. Since this study primarily depends on individuals’ self reported heath status and associated measurements, we accept the fact that variables may be associated with subjectivity and measurement error problems. To some extent, the use of mid-point income categories leads to loss of information. However, using all possible information seemed more appropriate than dropping the variables from estimations. In addition, our estimations are based on cross sectional data thus the estimated relationship may not reveal the most precise causality even after accounting for the effect of unobserved heterogeneity. In future, it is worthwhile to further investigate this issue with a longitudinal study provided that data can be gathered for all variables included in our estimations.

## 7. Conclusions and Policy Implications

Overall, this study suggests that not only do individually-centered socioeconomic conditions such as the level of education, income, age and risky behaviors contribute to the health of WV residents, but also that the surrounding economic environment can impact their health and quality of life. Findings from this study also provide evidence that urban sprawl is likely a contributing factor to lifestyle choices and, therefore, the health and obesity status of rural people.

In line with previous findings, this study also suggests that fruit and vegetable consumption is likely to lead to a lower level of obesity and better health. Therefore it is worthwhile to investigate programs which are in place to improve fresh fruit and vegetable production and their availability and affordability to rural residents. Policies which subsidize fresh fruit and vegetable production and that encourage fast food vendors to introduce more fruit and vegetable based items in their daily menus would be timely. In-kind subsidies to low income people to consume fruits and vegetables through welfare programs might also be another intervention strategy. In conjunction with policies which encourage consumers to choose less energy-intensive diets, the policy initiatives to encourage physical activities through better land use planning is also vital in controlling obesity in disadvantaged communities.

From an empirical point of view, it is necessary to address endogeneity and unobserved heterogeneity of health and behavioral outcomes to derive unbiased estimates. We believe that the significant methodological contribution of this study to the literature is in addressing the epidemiological black box which often omits the unobserved heterogeneous influences that could lead to spurious relationships.

Although there could be a bias associated in reporting self-assessed health, we believe that this study provides some useful insights to policy formulation in combating health issues like obesity and promoting well-being of residents of predominantly rural states. Toward this end, the results suggest that intervention strategies be targeted toward educational programs focusing on health, in conjunction with statewide income enhancing activities and careful land use planning.

## Figures and Tables

**Table 1.** Descriptive statistics for dependent, demographic, income and employment variables.

**Variable**	**Definition**	**Number**	**Mean/%**	**Min**	**Max**
	**Dependent Variables**				
	***Weight status***				
BMI	Body mass index*(kg/m^2^)	3236	27.42	13.76	62.78
OBESE	Obese a	896	27.69	0.00	1.00
	***Self perceived health status***				
OGENHLTH	Ordered health indicator (good=2, fair=1, poor=0)	3340	1.62	0.00	2.00
	Poor health	346	10.36	–	–
	Fair health	571	17.10	–	–
	Good health	2423	72.54	–	–
	**Covariates**				
	***Demographic categories***				
LEDUCA	Level of education (ordered categorical variable)**	3345	3.345	0.00	5.00
	Kindergarten or never attended	6.00	0.18	–	–
	Elementary school	232	6.94	–	–
	Some high school education	398	11.90	–	–
	High school graduate	1321	39.49	–	–
	Some college education	741	22.15	–	–
	College graduate	647	19.34	–	–
	***Gender***				
DSEX	Male = 1	1323	39.50	0.00	1.00
	***Ethnicity***				
MNONH	Multicultural non Hispanic	150	4.50	0.00	1.00
WNONH	White Non-Hispanic	3060	91.70	0.00	1.00
BNONH	Black Non-Hispanic	60	1.80	0.00	1.00
HISP	Hispanic	67	2.01	0.00	1.00
AGE	Age*	3349	51.00	18.00	97.00
PINC	Household Income *	2913	30460.01	7500.00	50000.00
	***Employment Status***				
OTHERE	other-employed	492	14.70	1.00	0.00
EMPLOYD	Employed	2051	61.26	1.00	0.00
STUDENT	Student	94	2.81	1.00	0.00
RETD	Retired	711	21.24	1.00	0.00

^a^ Obese indicator (=1 if BMI ≥ 30 kg/m^2^; 0 otherwise).

“*” designates continuous variables.

“**” indicates an ordinal variable. All other statistics represent the prevalence of respective categorical dummy variables.

**Table 2.** Descriptive statistics for marital status and other variables.

**Variable**	**Definition**	**Number**	**Mean/%**	**Min**	**Max**
	***Marital Status***				
WIDOW	Widowed	478	14.29	0.00	1.00
MALT	Married or cohabited	1914	57.22	0.00	1.00
DIVSEP	Divorced or separated	585	20.40	0.00	1.00
NMARRI	Never married	368	11.00	0.00	1.00
	**Other Covariates**				
SEDENT	Sedentary	421	12.81	0.00	1.00
SMOKING	Smoking	874	26.14	0.00	1.00
HCARE	Has health care	2794	83.63	0.00	1.00
RHEART	At risk of having heart problems	1251	37.43	0.00	1.00
RASTHMA	At risk of having asthma	300	8.98	0.00	1.00
RFDRHV	At risk of high alcohol consumption	89	2.68	0.00	1.00
FRTINDX	Fruit and vegetable index **	3349	2.70	1.00	4.00
DDISTD	Living in Depressed county	587	23.07	0.00	1.00
TRVT	Average travel time (minutes) to work *	2544	25.42	19.50	36.80

“*” indicates continuous variables All other statistics represent the prevalence of respective categorical dummy variables.

“**” indicates an ordinal variable.

**Table 3.** Maximum likelihood logit estimates of obesity risk and associated marginal probabilities.

**Variable**	**Definition**	**Estimate**	**Pr > ChiSq**	**Marginal effect**	
CONSTANT		−3.4410	0.0001	–	***
LEDUCA	Level of education	−0.1840	0.0008	−0.0344	***
WNONH	White Non-Hispanic	−0.2585	0.2597	−0.0483	
BNONH	Black Non-Hispanic	0.1662	0.6806	0.0311	
HISP	Hispanic	−0.8663	0.0609	−0.1620	*
PINC	Household income	0.0000	0.0204	0.0000	**
INCSQ	Household income squared	−0.0000	0.0081	0.0000	***
EMPLOYD	Employed	−0.2611	0.1138	−0.0488	
STUDENT	Student	−0.7912	0.0898	−0.1480	*
RETD	Retired	−0.2374	0.2822	−0.0444	
DSEX	Male	0.1726	0.1104	0.0323	
MALT	Married or cohabited	0.0359	0.8565	0.0067	
DIVSEP	Divorced or Separated	−0.2662	0.2244	−0.0498	
NMARRI	Never Married	0.3997	0.1137	0.0747	
AGE	Age	0.1409	0.0001	0.0263	***
AGESQ	Age squared	−0.0014	0.0001	−0.0003	***
SEDENT	Sedentary	0.5201	0.0015	0.0973	***
SMOKING	Smoking	−0.8086	0.0001	−0.1512	***
HCARE	Has health care	0.0330	0.8279	0.0062	
RFDRHV	At risk of alcohol consumption	0.0761	0.8211	0.0142	
FRTVINDX	Fruit and vegetable index	−0.1186	0.0655	−0.0222	*
DDISTD	Living in Depressed county	−0.0861	0.5035	−0.0161	
TRVT	Average travel time (minutes) to work	0.0218	0.0720	0.0041	*

*/**/*** Significant at 10%, 5%, or 1% or higher level, respectively. N = 2115

**Table 4.** Ordered logit estimates of self-assessed health.

Variable	Ordered Logit 1^a^		Ordered Logit 2^a^		Ordered Logit 3^a^	
	Estimate	Pr > ChiSq	Estimate	Pr > ChiSq	Estimate	Pr > ChiSq
Constant2	2.0358	0.0064 **	0.2509	0.6330 **	2.0418	0.0063 **
Constant1	3.6229	0.0001 ***	1.8296	0.0005 ***	3.6285	0.0001 ***
LEDUCA	0.1775	0.0061 ***	0.2614	0.0001 ***	0.1778	0.0061 ***
WNONH	−0.0782	0.7729	0.1359	0.6029	−0.0754	0.7806
BNONH	0.1278	0.7814	0.1192	0.7938	0.1343	0.7708
HISP	−0.4051	0.4088	0.1209	0.7949	−0.4036	0.4110
PINC	0.0000	0.0001 ***	0.0000	0.0001 ***	0.0000	0.0001 ***
EMPLOYD	1.4592	0.0001 ***	1.6625	0.0001 ***	1.4604	0.0001 ***
STUDENT	0.7726	0.1646	1.4885	0.0036 *	0.7691	0.1662 *
RETD	1.2294	0.0001 ***	1.5306	0.0001 ***	1.2285	0.0001 ***
DSEX	−0.0235	0.8571	−0.1047	0.4139	−0.0254	0.8460
MALT	−0.2083	0.2800	−0.3849	0.0412	−0.2143	0.2672
DIVSEP	−0.1369	0.5059	−0.1642	0.4282	−0.1404	0.4956
NMARRI	−0.0340	0.9048	−0.2828	0.3032	−0.0377	0.8947
AGE	−0.0298	0.0001 ***	−0.0248	0.0001 ***	−0.0301	0.0001 ***
PREDOBE	−2.8283	0.0003 ***	–	–	–	–
SEDENT	−0.5902	0.0008 ***	−0.8498	0.0001 ***	−0.5912	0.0008 ***
SMOKING	−0.8068	0.0001 ***	−0.4179	0.0021 ***	−0.8088	0.0001 ***
HCARE	−0.2290	0.1790	−0.2537	0.1363	−0.2291	0.1790
RHEART	−0.7851	0.0001 ***	−0.7779	0.0001 ***	−0.7610	0.0001 ***
RASTHMA	−0.8266	0.0001 ***	−0.8285	0.0001 ***	−0.8167	0.0001 ***
RFDRHV	0.0153	0.9695	0.0116	0.9769	0.0169	0.9663
FRTINDX	0.0563	0.4631	0.1298	0.0778	0.0576	0.4526
DDISTD	−0.4826	0.0002 ***	−0.4907	0.0001 ***	−0.4835	0.0001 ***
OBESE	–	–	−0.1798	0.1684	−2.8448	0.0003 ***
RES^a^	–	–			2.7328	0.0005 ***
−2LogL	2307.5760		2318.9260		2306.8640	

*/**/*** Significant at 10%, 5%, or 1% or higher level, respectively. N = 2101.

^a^ Ordered logit 2 and 3 are respective regressions used to test the endogeneity of obesity. Ordered logit 1 is self assessed health equation which is corrected for unobserved heterogeneity

^b^ RES is residual obtained form the 1^st^ stage estimation of obesity. The significant correlation of RES with health status in logit 3 implies that obesity is an endogenous variable correlated with unobserved factors related to health
